# Genetic diversity, reproductive capacity and alkaloids content in three endemic *Alkanna* species

**DOI:** 10.1371/journal.pone.0233516

**Published:** 2020-06-01

**Authors:** Ivanka Semerdjieva, Galya Petrova, Elina Yankova-Tsvetkova, Tsvetelina Doncheva, Nadezhda Kostova, Rozalia Nikolova, Valtcho D. Zheljazkov

**Affiliations:** 1 Department of Botany and Agrometeorology, Faculty of Agronomy, Agricultural University, Plovdiv, Bulgaria; 2 Department of Natural Sciences, New Bulgarian University Sofia, Sofia, Bulgaria; 3 Department of Plant and Fungal Diversity and Resources, Institute of Biodiversity and Ecosystem Research, Bulgarian Academy of Sciences, Sofia, Bulgaria; 4 Institute of Organic Chemistry with Center for Phytochemistry, Bulgarian Academy of Sciences, Sofia, Bulgaria; 5 Department of Crop and Soil Science, Oregon State University, Corvallis, Oregon State, United States of America; Institute for Biological Research "S. Stanković", University of Belgrade, SERBIA

## Abstract

The Balkans endemic species *Alkanna primuliflora* Griseb., *A*. *stribrnyi* Velen., and *A*. *graeca* Boiss. & Spruner have limited distribution in the Balkan Peninsula and a large variation in the morphological characteristics. The populations of the three *Alkanna* species in the Bulgarian flora are small and fragmented. There are no previous reports on the chemical profile or on the embryology of these species. The hypothesis was that the limited distribution of *A*. *primuliflora*, *A*. *stribrnyi*, and *A*. *graeca* was due to their reproductive capacity and genetic diversity. Furthermore, we hypothesized that the three species will contain pyrrolizidine alkaloids (PAs), as other species of the genus *Alkanna* (Boraginaceae), but they would have differential alkaloids composition. The population genetic structure and differentiation showed a clear distinction between species and revealed average levels of genetic diversity among the natural populations of the three *Alkanna* species. The embryological investigation observed stability of the processes in the male and female generative spheres and high viability of mature pollen and embryo of the three species. The normal formation of male and female gametophytes without deviations or degenerative processes, and observed levels of genetic diversity between *Alkanna* individuals are important in maintaining the size and resilience of the *Alkanna* populations. Eight alkaloids were identified by GC-MS in *A*. *primuliflora* and *A*. *graeca* and six alkaloids in *A*. *stribrnyi*. The main pyrrolizidine alkaloids (PAs) in all investigated species was triangularine. *A*. *primuliflora* and *A*. *graeca* showed similar chemical composition that comprised 9-angeloylretronecine, 7-tigloylretronecine, 9-tigloylretronecine, triangularicine, dihydroxytriangularine, dihydroxytriangularicine, whereas, in *A*. *stribrnyi* 9-tigloylretronecine, triangularicine and dihydroxytriangularicine were not found. This is the first report on the presence of PAs in *A*. *primuliflora*, *A*. *stribrnyi* and *A*. *graeca*. Besides, this is the first report on the embryology of these endemic species. The results contribute to the knowledge of the three endemic *Alkanna* species and will facilitate policy-making and defining new strategies for their conservation.

## Introduction

The genus *Alkanna* belongs to family Boraginaceae, subfamily Boraginoidae, tribe Lithospermeae [1]. The *Alkanna* species show the greatest diversity in the southern part of the Balkan Peninsula, in the Mediterranean and Irano-Turanian region, and subtropical areas of the world [2]. Generally, genus *Alkanna* is represented by a large number of local and regional endemic species [3]. Six *Alkanna* species are found in the Bulgarian flora and five of them are either Balkan or Bulgarian endemics. *Alkanna primuliflora* Griseb., *A*. *stribrnyi* Velen. and *A*. *graeca* Boiss. & Spruner are Balkan endemics, while *A*. *stojanovii* Kožuharov and *A*. *jordanovii* Kožuharov are two Bulgarian endemics [4]. The three studied species have very limited distribution in Bulgaria as follow: *A*. *graeca*—in two floristic regions, Pirin Mt (South), Thracian Lowland; *A*. *stribrnyi*–in three floristic regions, Slavyanka Mt, Thracian Lowland, Pirin Mt (South); and *A*. *primuliflora*—mainly in four floristic regions, Thracian Lowland, Pirin Mt (South), Valley of Struma river, and Slavyanka Mt. The populations of *A*. *primuliflora*, *A*. *stribrnyi*, and *A*. *graeca* are included in the European ecological network EU NATURA 2000 under Directive 92/43 / EEC on Habitats. In Bulgaria, the populations of *Alkanna* species are distributed on dry, rocky places. Generally, the populations of three *Alkanna* species are small, fragmented and represented by a very limited number of individual plants.

Overall, there is a large variation in the morphological characteristics between the *Alkanna* species but also within a species. Some variations include variously colored corolla in yellow (*A*. *primuliflora)*, orange (*A*. *stribrnyi*) or yellow or orange (*A*. *graeca*), with more or less cylindrical tube and infundibuliform limb, with a ring of hairs [4]. *Alkanna primuliflora*, *A*. *stribrnyi* and *A*. *graeca* are perennial, pubescent herbaceous plants. They are insect-pollinated, they flower in April-May, and are seed propagating plants [4]. Morphological traits of taxonomic importance include color of the corolla and nutlets, shape and size of calyx, leaf form and trichomes [4,5]. The trichomes in the leaf and other parts of *Alkanna* species may be glandular or non-glandular [4,6]. According to Kožuharov [4], the three *Alkanna* species prefer dry and hot places, and they are found on rocky slopes of up to 900 m asl. *Alkanna stribrnyi* and *A*. *graeca* have transient morphological characteristics with those of *A*. *primuliflora* [4].

There are no comprehensive phylogenetic studies on the genus *Alkanna*, although several molecular studies have been published [7–10]. However, the latter reports described studies on the evolutionary history and phylogenetic relationships in the Boraginaceae family, especially on taxa belonging to Lithospermeae and Boragineae [7–11].

The DNA and ISSR analysis can be additional keys to describe and differentiate the *Alkanna* species. Also, the content of alkaloids in various *Alkanna* species can be used as a chemotaxonomic marker. The value of chemotaxonomic markers in the systematics of the family Boraginaceae has been elaborated previously [12,13]. Plants of the family Boraginaceae are known for the synthesis and accumulation of pyrrolizidine alkaloids (PAs) [14]. This class of alkaloids received considerable attention because of their hepatotoxic, mutagenic and cancerogenic activities and this class of alkaloids has been widely used as a chemotaxonomic marker [12, 15–19]. The PAs have been previously documented only in two *Alkanna* species; *A*. *orientalis* and *A*. *tinctoria* [20–25].

*Alkanna primuliflora*, *A*. *stribrnyi* and *A*. *graeca* have limited distribution in the flora of the Balkans. Generally, there are several major reasons for the limited distribution of plant species, and these include; (1) habitat preferences; (2) poor competitiveness; (3) problems in the reproductive potential of the species. Investigation of the peculiarities in the generative sphere in a particular plant species is a key factor in assessing the species reproductive potential. The features of reproductive biology in studied species (processes of development of gametes, pollination, the formation of embryo and endosperm, pollen and seed vitality) determining their reproductive potential, can provide important information on the factors limiting or preventing the reproduction of a specific species. The study of the biology of reproduction together with the determination of the genetics of their populations will help to define the causes for the limited distribution of the three *Alkanna* species. Such studies in combination with pollination studies, would facilitate defining strategies for plant species conservation (e.g. *in vitro* propagation, ex-situ collection). Due to the limited distribution of endemic *Alkanna* species, there are no reports on pyrrolizidine alkaloids content in *A*. *primuliflora*, *A*. *stribrnyi*, and *A*. *graeca*. Embryological studies also were not previously conducted on the three targeted species. In addition, no comparative study on the three *Alkanna* species embryology was published. Therefore, the purpose of this study was to: (1) distinguish and detect molecular genetic markers between *A*. *primuliflora*, *A*. *stribrnyi*, *A*. *graeca*; (2) reveal the alkaloids composition; (3) reveal embryological features that may be contributing to the limited distribution of these species.

The hypothesis was that the limited distribution of *A*. *primuliflora*, *A*. *stribrnyi*, and *A*. *graeca* was due to their reproductive capacity and genetic diversity. Furthermore, we hypothesized that the three species will contain pyrrolizidine alkaloids (PAs), as other species of the genus *Alkanna* (Boraginaceae), but they would have differential alkaloids composition.

The alkaloid profile of the investigated species could also provide supplementary information for the species diversity. However, the application of alkaloids for this purpose has to be cautiously used, because the alkaloid pattern is influenced by the environmental factors. Therefore, the alkaloid profile of the species was compared at two levels, between different species and within the same species in different natural localities or stage of development. The Ministry of Environment and Water of Bulgaria could use the result from this study and consider these endemic species for a protection status.

## 2. Materials and methods

### 2.1. Plants material

The plant materials for this study were *Alkanna primuliflora* Griseb., *A*. *stribrnyi* Velen., and *A*. *graeca* Boiss. & Spruner. The five collection locations of *Alkanna* species with the exact coordinates, altitude, and habitats are presented in Table 1. Voucher specimens of *A*. *primuliflora*, *A*. *stribrnyi*, *A*. *graeca* were deposited at the Herbarium of the Agricultural University, Plovdiv, Bulgaria (SOA) [26].

**Table 1:** Collection sites, location coordinates, altitude (m.a.s.l.) and habitats of *Alkanna primuliflora*, *A*. *stribrnyi* and *A*. *graeca* in Bulgaria.

Species	Location and habitat	GPS coordinates masl
*A*. *primuliflora*	Thracian lowland, Plovdiv, Sienite rocks, dry places (Aprm1, in flowering); (Aprm2, after flowering)	42°08'16.9"N 024°43'63.8"E 107masl
*À*. *stribrnyi*	Thracian lowland above Asenovgrad town, Sv. Petka, dry stone places, metamorphic rocks, The soil cover is very thin (Ast1)	41°59'35.8" N 024°52'20.1" E 310masl
	Thracian lowland, above Asenovgrad town, Asenova kr, dry stone places, metamorphic rocks, The soil cover is very thin. (Ast2)	41°59'51.8" N 024°52'53.1" E 418masl
	Thracian lowland, Novo selo villige, karst marbles and gabbro-diorite covered with thin lake Pliocene sediments. The soil cover is very thin. Almost completely deforested. (Ast3)	42°05'39.9"N 024°28'01.1"E 328masl
*A*.*graeca*	Above Asenovgrad town, dry stone places, metamorphic rocks, The soil cover is very thin. (Agr1)	41°59'13.8" N 024°52'17.1"388masl

*A*. *primuliflora*—Aprm1 (population 1, in flowering); Aprm2 (population 1, after flowering); *A*. *stribrnyi* -Ast1 (population 1, in flowering); Ast2 (population 2, in flowering), Ast3 (population 3, in flowering); *A*.*graeca*–Agr 1 (population 1, in flowering).

### 2.2. Methods

Plant samples were taken separately for DNA, phytochemical, and embryological studies. Plants were sampled for DNA, alkaloid, and some embryological analyses in May. Only plant leaves were collected for the DNA analyses, while whole plants were collected for the alkaloids testing. For the embryology analyses, flower buds and open flowers were collected in May and then seeds were collected in June-July.

#### 2.2.1. DNA extraction

This study attested that the individual plants within populations of the three *Alkanna* species in Bulgaria had a very limited number. Therefore, leaf samples were collected carefully from a few plants to minimize the potential damage to the populations. We performed the sampling depending on the density of each population; plants were randomly chosen for sampling. For genetic analysis, we used the leaves of the three species at flowering (in May) as follow: *A*. *primuliflora*–ten samples; *A*. *graeca*–eight samples; *A*. *stribrnyi*–six samples. Тhese are the total number of samples per species per population as shown in Table 1.

The DNA from the three endemic species was extracted using the standard CTAB extraction procedure [27] with minor modifications. The modified method involves isolation of gDNA by a modified-CTAB method employing a high concentration of PVP at around 2.5% w/v and 2-BME up to 5% v/v. DNA quality and concentrations were measured using a NanoDrop ND-1000 spectrophotometer (Saveen and Werner AB, Limhamn, Sweden). DNA samples were diluted to 25 ng/μl and stored at -20°C before amplification.

#### 2.2.2. Inter-simple sequences repeat (ISSR) amplification product analysis

First, the polymorphism of 40 markers was tested and then 10 polymorphic and reproducible ISSR primers (Microsynth, Balgach, Switzerland) were selected and used (Table 2). Polymerase chain reactions (PCR) were performed according to the method described previously [28]. PCR reactions were carried out in a Techne TC-5000 gradient thermal circler (Techne, Staffordshire, UK). The reproducibility of the technique was tested by replicating each amplification reaction twice. Amplification products were separated on 2% agarose gels stained by incorporating 1% GelRed (Biotium Inc., USA) at 1.5h, 135V along with 100 bp Plus DNA Ladder (Thermoscientific, Vilnius, Lithuania). The DNA fragments were visualised under UV light and further analysed with a video image analyser (BioImaging Systems, Cambridge, UK).

**Table 2:** ISSR primers used for analysis of genetic diversity of *Alkanna primuliflora*, *A*. *stribrnyi* and *A*. *graeca* in Bulgaria.

Primer	Sequence 5'- 3'	Total number of bands	Number ofpolymorphic bands	Polymorphism (%)	Annealing temperature(°C)
ISSR1	(CA)_8_G	12	7	58	55
ISSR2	(AC)_8_C	14	14	100	55
ISSR3	(AC)_8_G	13	10	77	55
ISSR4	(AG)_8_YC	10	7	70	60
ISSR5	(AC)_8_YT	14	14	100	60
ISSR6	(AC)_8_YG	9	4	44	60
ISSR7	(AG)_10_C	10	8	80	60
ISSR8	(AG)_8_YT	12	8	67	60
ISSR9	(AC)_8_YA	12	5	42	60
ISSR10	(AC)_8_YG	9	7	78	60
Total		115	85	72	

P: % of polymorphic loci; *He*: expected heterozygosity, *uHe*: unbiased expected heterozygosity; SI: Shannon’s information index; Standard error is shown in parentheses.

#### 2.2.3. ISSR data analysis

To construct binary matrix the ISSR bands were scored as presence (1) or absence (0) of binary characters. The matrix was used for calculation of population genetic variation indices and assessment of the genetic diversity and structure of the *Alkanna* populations. The following genetic diversity indexes were calculated to determine the level of genetic variation within populations: the percentage of polymorphic loci (*P*), expected heterozygosity (*He*), unbiased expected heterozygosity (*uHe*) and Shannon’s information index (*SI*) using GenAlEx *ver*. 6.5 [29]. Also, Principal Coordinate Analysis (PCoA) and Hierarchical Analysis of Molecular Variance (AMOVA) in GenALEx software were conducted to show the genetic relationships among investigated *Alkanna* populations based on Nei’s genetic distances. Bayesian clustering-based structure analysis was performed on the entire data set in STRUCTURE *ver*. 2.3.4), which uses a Markov Chain Monte Carlo (MCMC) algorithm to detect population structure [30]. To estimate the admixture of individuals into groups (K) and the best K value (number of groups), the Evanno test was performed on STRUCTURE results using STRUCTURE HARVESTER *v*.0.6.8 [31,32]. Finally, genetic distances between all *Alkanna* individual plants were calculated based on the results of Nei’s genetic distance [33].

#### 2.2.4. Extraction of plant material and qualitative analysis of alkaloids

The plant materials used included above ground plant parts of at least 10–15 randomly selected plants (per species) of *A*. *primuliflora* (Aprm1- 24.17g), *A*. *stribrnyi*, (Ast1- 14.29g), (Ast2- 6.89 g) (Ast3- 11.46g) and *A*. *graeca* (Agr1- 14.41g) in flowering period and *A*. *primuliflora* (Aprm2- 5.08g) in after flowering period from the five collection locations (Table 1). The plant material of each sample was extracted exhaustively with CH_3_OH in a Soxhlet apparatus. The combined CH_3_OH extracts after evaporation to dryness were acidified with 5% HCl, filtered and extracted with CHCl_3_. The aqueous acid solutions were stirred with zinc (Zn) dust (24 h) then filtered and made alkaline with 25% NH_4_OH to pH 9. The alkaline solutions were extracted with CHCl_3_ to give crude alkaloid mixtures (CAM (Apr1)—15.61 mg; CAM (Aprm2)—4.67 mg; CAM (Ast1)—10.76 mg; CAM (Ast2)—6.1 mg; CAM (Ast3)—9.15 mg and CAM (Agr1)—12.27 mg). The crude mixtures of alkaloids were analyzed by Gas chromatography-mass spectrometry (GC/MS). The alkaloids 1 to 8 were identified by comparison of their mass spectral fragmentation with standard reference spectra from database Wiley 275 and the literature data [13,34]. The GC/MS analysis was performed with GC Hewlett Packard 6890 (Palo Alto, California, U.S.A.) using MS detector Hewlett Packard 5973 fitted with an HP-5 MS column (30 m x 0.25 mm x 0.25 μm). The program was as follows: injector T 250°C; the temperature program was 100°C (2 min) to 280°C, 5°C/min, isothermal at 280°C for 20 min. Split ratio 1: 30, carrier gas (He), constant flow of 0.8 mL/min.

#### 2.2.5. Embryological analyses

The major parameters of the reproductive biology of the studied species (structures and processes in the generative sphere, pollen and seed viability) were investigated to estimate their reproductive capacity. For the embryological studies we used flowers, buds and open flowers in different developmental stages collected from 30 individuals of each population. The material (flower buds and open flowers) was fixed in a mixture FAA (formalin: glacial acetic acid: 70% ethanol in a ratio of 5:5:90 parts, respectively). Then the fixed plant material was treated according to the classical paraffin methods [35] to obtain permanent microscopic slides. Heidenhain’s haematoxylin as colouring agent [36] and Entellan Merck (rapid non-aqueous mounting medium, containing xylene) as a slide mounting medium were used for the permanent slides. The main embryological structures and processes in the male and female generative spheres were established after observations on Olympus Light CX2 microscope (Olympus Corporation, Shinjuku, Tokyo, Japan). The microphotographs were prepared using “Infinity lite” digital camera 1.4 Mpx.

*2*.*2*.*5*.*1*. *Polen viability*. Assessment of the pollen viability was conducted using Acetocarmine test [37]. According to the method used, temporary slides were prepared after staining the pollen grains. The pollen grains from 30 anthers from different individual plants per species were counted. Before that, the pollen grains were coloured with a solution containing 1% acetocarmine and were stained in red (viable, fertile) and unstained (nonviable, sterile). The mature pollen grains were counted (in the visual field at 100x magnification) using a light microscope.

*2*.*2*.*5*.*2*. *Seed viability testing*. The tetrazolium test was used for estimation of the viability of seeds. One hundred seeds per population of *A*. *primuliflora*, *A*. *stribrnyi*, and *A*. *graeca* were used. The seeds from different individual plants per tested species were sampled at maturity of the three species, which occurred in June–July. Before treatment with 1% solution of the tetrazolium chloride, the seeds were kept for 24 hours in Petri dishes on wet filter paper at 25°C temperature. The seeds were incubated in 1% solution of 2, 3, 5-triphenyltetrazolium chloride according to the methods outlined in AOSA (Association of Official Seed Analysts) [38]. Initially, the tetrazolium solution is colourless, but later on, it changes from dark pink to red as a result of the action of hydrogen ions coming from the respiration process of the seeds. Embryos that show a physiological activity (active respiration) turn red and are therefore considered viable. The darker the red colour, the greater the respiratory activity of the seeds.

## 3. Results

### 3.1. Population genetic diversity study

Of the 40 ISSR primers used for polymorphism validation, 10 primers were polymorphic and up to 115 reproducible bands (all species together) were generated, and 85 (73.9%) of those, were polymorphic (Table 2). The size of all bands ranged from 100 to 3000 bp and the number of bands by each primer was from 9 to 14 with an average of 11.5 per primer (Table 3). The percentage of polymorphic loci (*P*) varied from 41.7% in *A*. *stribrnyi* to 64.6% in *A*. *graeca*. *Alkanna graeca* exhibited the highest values of genetic indexes expected heterozygosity *(He)*, unbiased expected heterozygosity *(uHe)* and Shannon’s information index *(SI)* of 0.157, 0.167 and 0.256, respectively. The investigated *Alkanna* species had a total of 20 private alleles, nine of *A*. *primuliflora* and *A*. *graeca* and two of *A*. *stribrnyi* (Table 3).

**Table 3:** Genetic diversity indices of investigated *A*. *primuliflora*, *A*. *stribrnyi* and *A*. *graeca*.

Population	*P* (%)	*He*	*uHe*	SI	Private bands
*Alkanna primuliflora*	62.5	0.137(0.022)	0.144(0.023)	0.227(0.032)	9
*Alkanna graeca*	64.6	0.157(0.022)	0.167(0.024)	0.256(0.033)	9
*Alkanna stribrnyi*	41.7	0.147(0.028)	0.161(0.031)	0.220(0.041)	2
Mean (SE)	56.3(7.32)	0.147(0.014)	0.157(0.015)	0.234(0.020)	6.67

#### 3.1.1. Population genetic structure and differentiation

**Fig 1:**
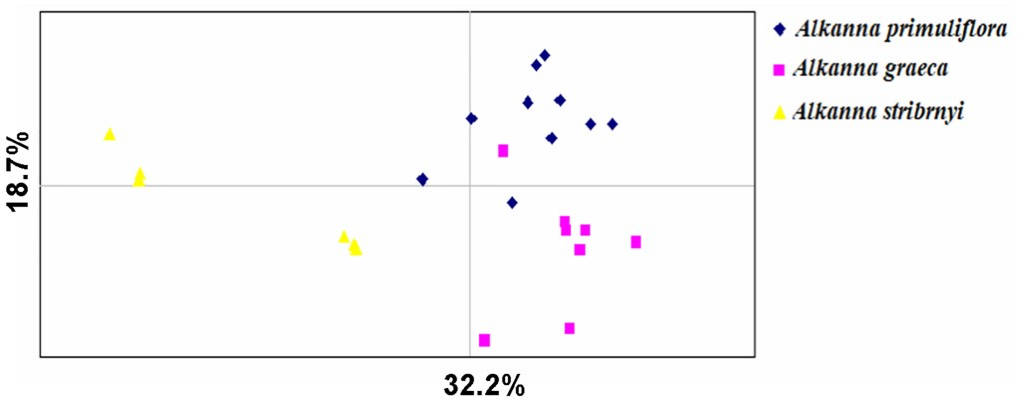
A two-dimensional PCoA plot of the all analyzed individuals of *Alkanna primuliflora*, *A*. *graeca* and *A*. *stribrnyi* in Bulgaria. The first and second principal coordinates account for 32.2% and 18.7% of the total variation, respectively.

In the two-dimensional PCoA, the results showed a clear differentiation between the species; the first two principal components explained 18.7% and 32.2% of the total variance, respectively (Fig 1). Here, the individuals clustered strongly according to their species assignation and form separate groups. For example, individuals from *A*. *primuliflora* and *A*. *graeca* form two separate clusters that combined overlap only a few individuals. *Alkanna stribrnyi* clustered separately and some individuals fell quite isolated from each other and the other populations.

The individuals of populations were able to form clear clusters, according to their geographical location, which suggests a strong genetic structure of populations studied herein. Only a few *A*. *graeca* individuals fell into *A*. *primuliflora* population cluster. This result could be explained with the presence of overlapping areal between natural *Alkanna* populations.

**Fig 2:**
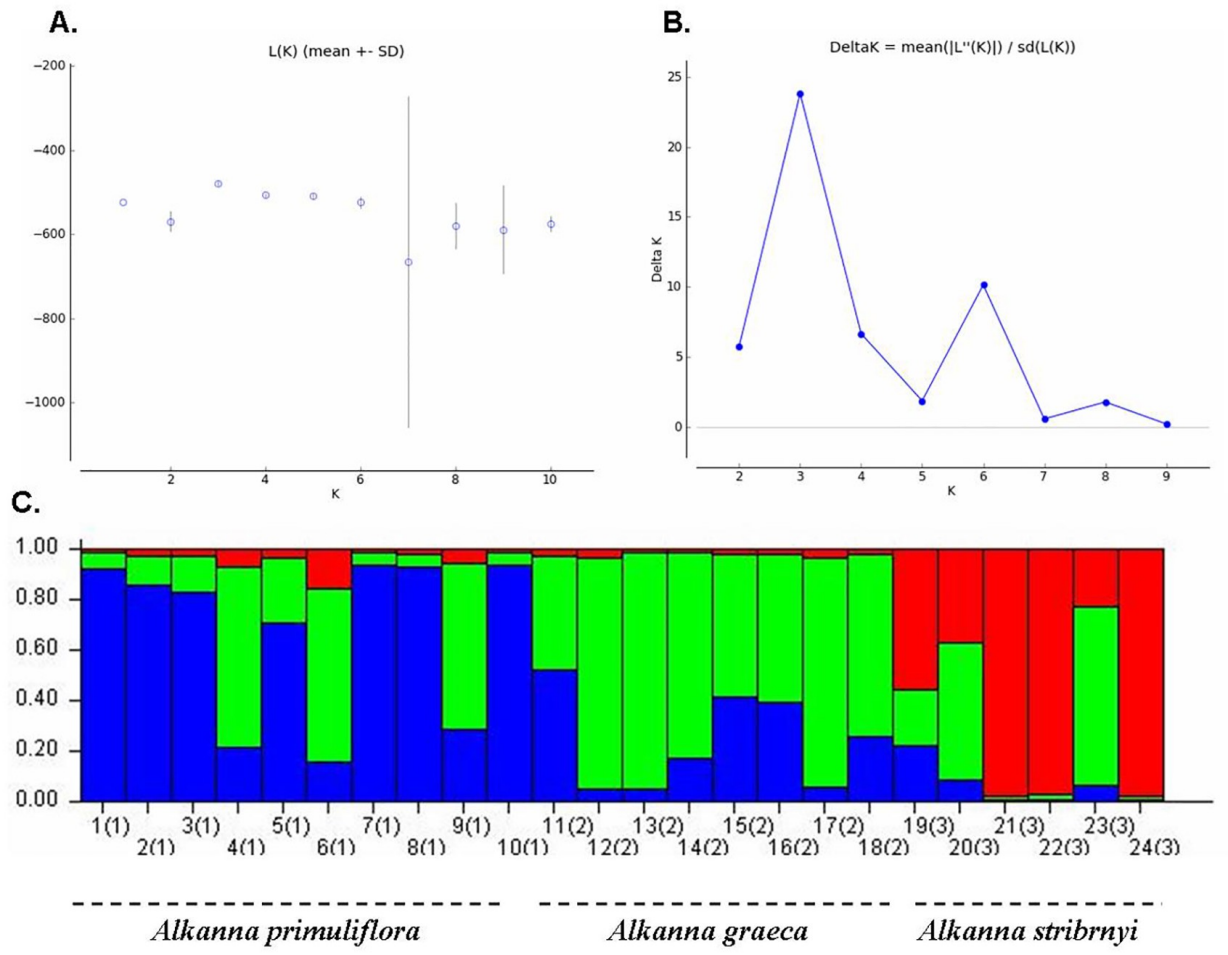
(A) Plot of K clusters versus mean (±SD) likelihoods (L[K]): (B). K plotted against the second order rate of change of the likelihoods (ΔK); (C). Genetic relationships among the populations of *Alkanna* estimated using STRUCTURE program based on ISSR data. STRUCTURE clustering results for K = 3 to as suggested in (B). Estimated genetic clustering obtained with the STRUCTURE for all investigated *Alkanna* individuals.

Population genetic structure was further studied using a Bayesian clustering algorithm, perform in STRUCTURE (Fig 2).

**Fig 3:**
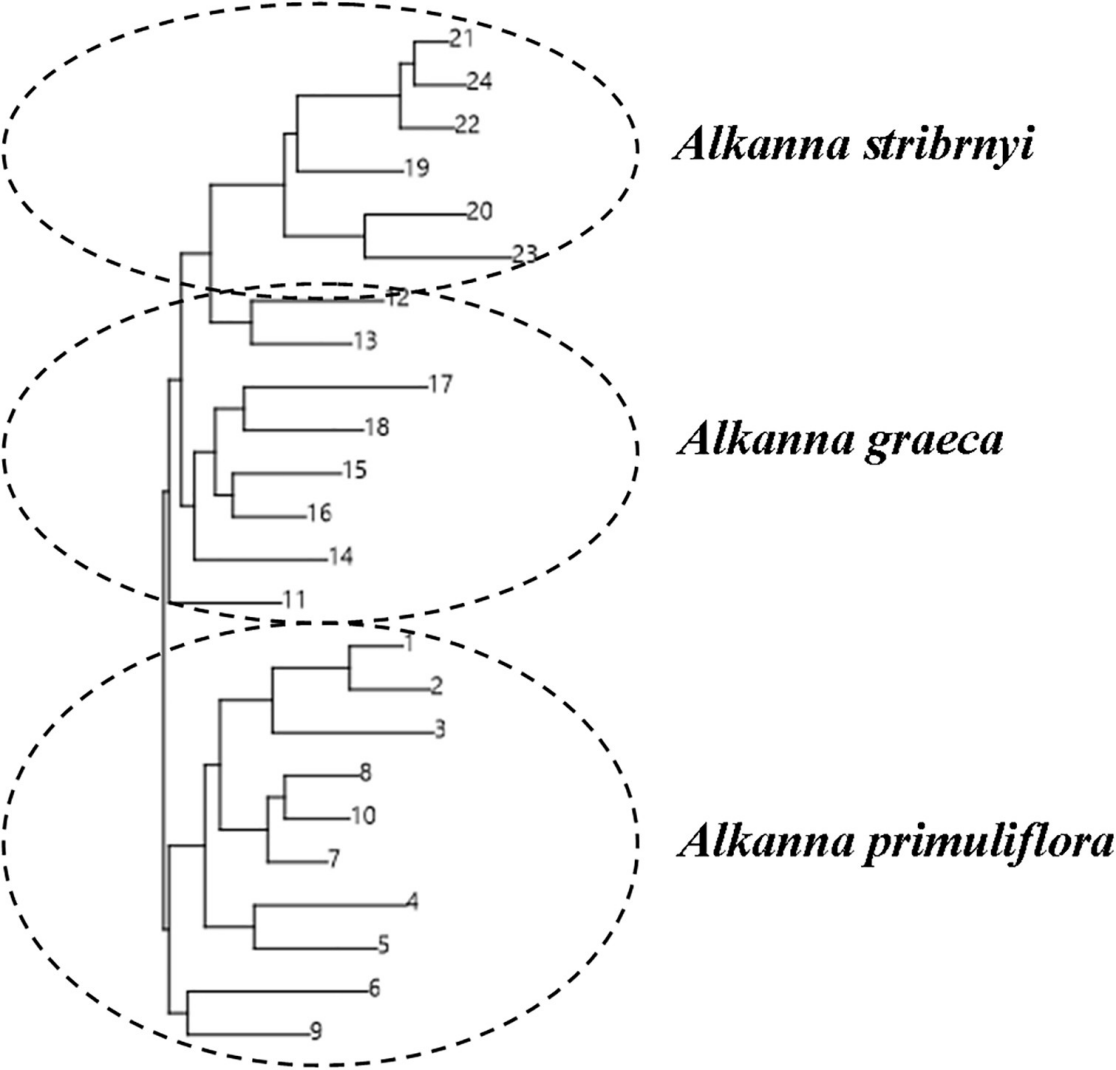
Graphical representation of hierarchical cluster analysis by UPGMA method based on Nei’s genetic distances among *Alkanna* individuals obtained by ISSR genetic markers.

According to the method described by Evanno et al. [31], the optimal cluster number was K = 3 (Fig 2A and 2B). The genetic differentiation was determined by hierarchical AMOVA and showed significant genetic differences among the populations of the three species; 71% of the variation resided within populations and showed that genetic differentiation was predominantly between individuals of each population (Table 4). Besides, we performed genetic relationship analysis among populations with neighbor-joining criteria based on the results of Nei’s genetic distance. The three *Alkanna* populations were divided into three main clusters, which corresponded to the geographic population areas (Fig 3).

**Table 4:** Analysis of molecular variance based on the ten ISSR markers for the *Alkanna* populations.

Source of variance	df	*SS*	MS	Variance component	Percentage total (%)	*F* statistic	*P*
**Among populations**	2	44.025	22.013	2.138	29	*F*_PT_ = 0.203	**0.001**
**Within populations**	21	110.58	5.265	5.265	71	*F*_PT_ = 0.289	**0.001**
**Total**	**23**	**154.583**		**7.403**	**100**		

* df-Degree of freedom, SS-sum of squares, MS-expected mean squares; P-value denotes the probability of null hypothesis.

### 3.2. Determination of alkaloid content

This study of the three *Alkanna* species determined eight alkaloids of pyrrolizidine type (Table 5). Eight alkaloids were determined in *A*. *primuliflora* and *A*. *graeca*, and six alkaloids in *A*. *stribrnyi*. The main alkaloid in *Alkanna* species was triangularine (5). The *A*. *primuliflora* plants at both development stages (Aprm 1-in flowering and Aprm 2 –after flowering) and *A*. *graeca* had more complicated alkaloid profile than *A*. *stribrnyi* due to the presence of a larger number of alkaloids. The plants of *A*. *stribrnyi* in all three populations (Ast1, Ast2, Ast3) had a similar alkaloid composition. The differences were only in the population (Ast3), where alkaloid dihydroxytriangularine (7) was absent and 9-tigloylretronecine (4) was present in the alkaloid mixture (Table 5).

**Table 5:** Alkaloids identified from *A*. *primuliflora*, *A*. *graeca*, *and A*. *stribrnyi* from different natural populations.

Alkaloids/species	*A*. *primuliflora*		*A*. *graeca*	*A*. *stribrnyi*		
	(Aprm 1)	(Aprm 2)	(Agr 1)	(Ast1)	(Ast2)	(Ast3)
7-Angeloylretronecine (**1**)	+	+	+	+	+	+
9-Angeloylretronecine (**2**)	+	+	+	+	+	+
7-Tigloylretronecine (**3**)	+	+	+	+	+	+
9-Tigloylretronecine (**4**)	+	+	+			+
Triangularine (**5**)	++	++	++	++	++	++
Triangularicine (**6**)	+	+	+			
Dihydroxytriangularine (**7**)	+	+	+	+	+	
Dihydroxytriangularicine (**8**)	+	+	+			

+presence of alkaloid; ++ dominant alkaloid; *A*. *primuliflora*—Aprm1 (population 1, in flowering); Aprm2 (population 1, after flowering); *À*. *stribrnyi*–Ast1 (population 1, in flowering); Ast2 (population 2, in flowering), Ast3 (population 3, in flowering); *A*.*graeca*—Agr 1 (in flowering).

### 3.3. Embryological research

This embryological study showed the main characteristics of the structures and processes in the male and female generative sphere and revealed similarities between the three endemic *Alkanna* species.

#### 3.3.1. Male generative sphere

**Fig 4:**
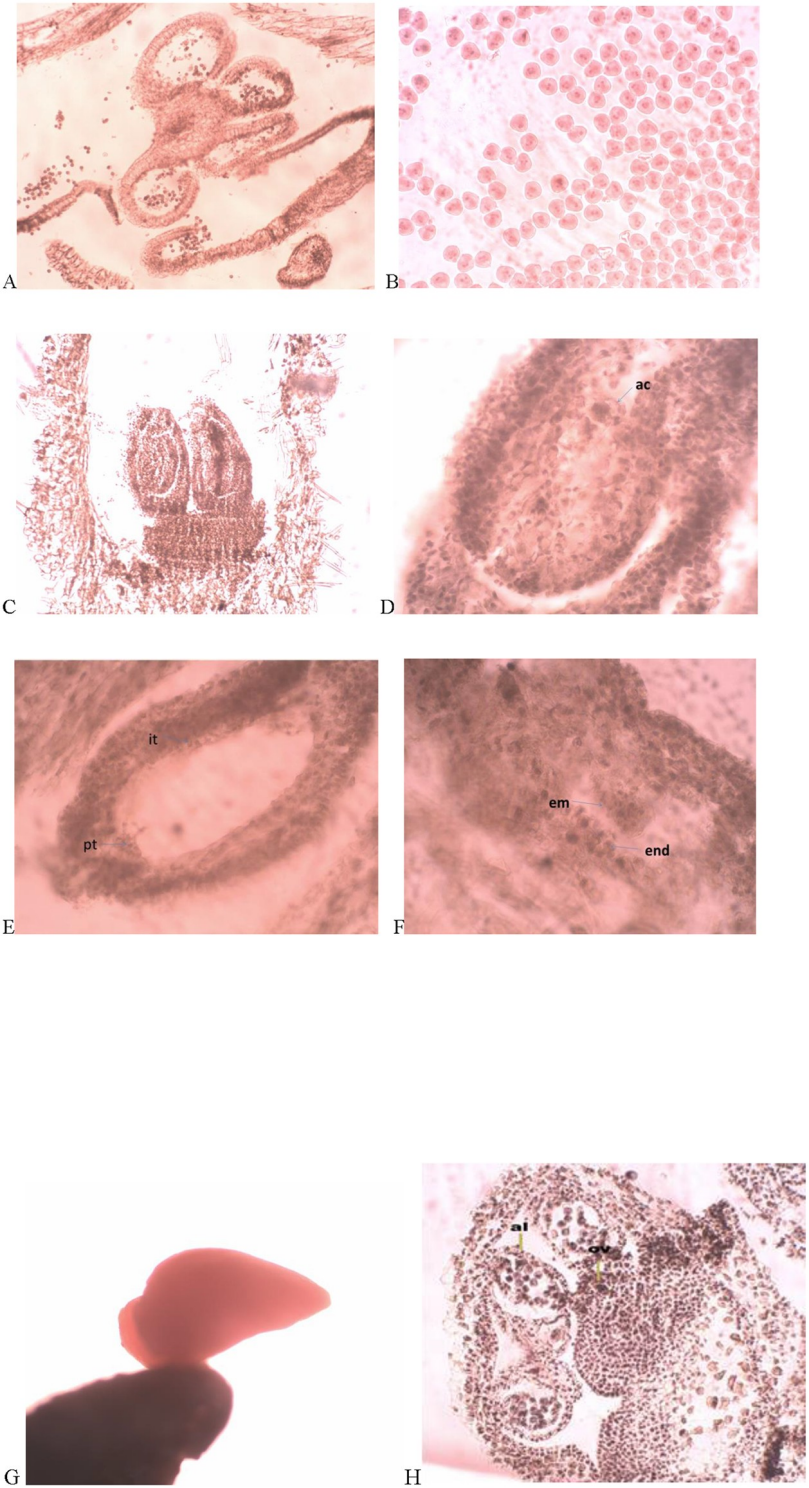
Pecularities of the reproductive sphere of studied *Alkanna* species. (A) tetrasporangite anthers in *A*. *primuliflora*; (B) two-celled mature pollen grains in *A*. *primuliflora*; (C) two-loculate ovary in *A*.*stribrnyi*; (D) anatropous ovule with one-celled archesporium in *A*. *primuliflora*; (E) mature embryo sac with postament in the chalazal part in *A graeca*; (F) globular embryo in *A*.*sribrnyi*; (G) mature embryo with part of endosperm and seed coat in *A*. *primuliflora*; (H) proterandry in *A*. *primuliflora* (x40 for Fig H); (x100 fpr Figs A,B,C,G), (x400 for Figs D,E,F); **ac**- archesporium cell, **it**-integumentary tapetum, **pt**-postament, **em**-embryo, **en**-endosperm, **ov**-ovule, **al**-anther locule.

The characteristics of the male generative sphere were: tetrasporangiate anthers (Fig 4A), four-layered anther wall developing after Dicotyledonous-type [39] and multilayered sporogenous tissue. The anther wall consisted of the epidermis, fibrous endothecium, one ephemeral middle layer and glandular tapetum. At the beginning of anther ontogenesis, the sporogenous cells were polygonal and fit closely with each other. Lather on, they elongate, rounded up and differentiated into microspore mother cells (MMCs) from which after simultaneous microsporogenesis predominantly tetrahedral microspore tetrads were formed. The mature pollen was 2-celled (Fig 4B).

#### 3.3.2. Female generative sphere

The female generative sphere consisted of a two-locular ovary with one anatropous tenuinnucelate ovule in each locule (Fig 4C). *A*fter meiosis in the ovule, one-cellulate archesporium formed hypodermal layer (Fig 4D). The embryo sac developed according to Polygonum—(monosporic)—type. From the specialized ovule structures in the three *Alkanna* species were established integumental tapetum and postament (Fig 4E). The embryo development follows the Asterad-type. The endosperm formation passes a nuclear stage and transforms into cellular at the globular embryo stage (Fig 4F). The mature embryo was straight and occupied ¾ parts of the seed (Fig 4G). A proterandry was established, when in the anthers one-celled pollen grains were formed, in the ovule macrospore mother cell was observed (Fig 4H). During the study, apomixis was not registered.

#### 3.3.4. Pollen and seed viability

After the application of acetocarmine stain technique for estimation of the pollen viability, cytoplasm and nuclei of the viable pollen grains were stained in red (Fig 4B). The results of this study showed high viability of the mature pollen in the populations of the three *Alkanna* species: 96.4% in the population of *A*. *primuliflora*, 89.5% in the population of *A*. *stribrnyi* and 88.9% in the population of *A*. *graeca*. The tetrazolium test showed viable seeds (embryos) (Fig 4G) that was estimated as follows: 50% in *A*. *primuliflora* 54.2% in *A*. *stribrnyi* and 56.1% in *A*. *graeca*.

## 4. Discussion

Information on the current status and the conservation of plant habitats, as well as the limited distribution of endemic species, is important for ecologists, conservationists and public institutions. In this respect, the main objective of the European ecological network European Union (EU) NATURA 2000 and Directive 92/43 / EEC on Habitats is the conservation of wild flora and fauna (https://ec.europa.eu/environment/index_en.htm). The initiative covers around 18% of the EU’s land area, as well as 6% of the EU marine territory. Therefore, Natura 2000 is the largest coordinated network of protected areas in the world. This document and the initiatives require special measures to be taken to ensure the sustainability of the populations and conservation of species. As a basis for determining biodiversity conservation measures and strategies, it is necessary to know: (1) the biology and ecology of the species; (2) the species reproductive capacity; and (3) the species genetic diversity. The study of reproductive characteristics and abilities is essential in the case of endemic and restricted species [40].

Although the observed habitats of three *Alkanna* species in Bulgaria were small, fragmented, and with a limited number of individual plants, this had no effect on the reproductive structures and capacity of the plants. The peculiarities of the reproductive biology of the three species revealed in the study, characterized them as strictly sexually reproducing species (no evidence of apomictic development was observed). This study also established the stability of the processes in the male and female generative spheres such as the normal formation of male and female gametophytes without deviations and degenerative processes. Because, from the tree analysis *A*. *graeca* and *A*. *stribryni* seem closer, since they share a branch in contrast to the PCoA where *A*. *graeca* and *A*. *primuliflora* are closer, we can assume the existence of hybrid individuals. In general, we hypothesize that the opportunity for hybridization exists between the *Alkanna* species studied here. The sexual reproduction by hybridization could further contribute to the genetic diversity of the three *Alkanna* species. It also increases the species’ ability to adapt to climate change. In agreement with this, there is an overlap of blooming period and pollinators in sympatric areas of studied *Alkanna* species, which potentially favour pollen exchange between species. In comparison with other *Alkanna* species e.g. *A*. *orientalis* [41], this study revealed average levels of genetic diversity among the natural populations of the three *Alkanna* species. The observed levels of genetic diversity between *Alkanna* individuals in this study can be considered a positive indicator for the population viability. Furthermore, the estimated high pollen and embryo viability play an important role in maintaining the size and resilience of populations of the three *Alkanna* species. The proterandry in the studied species revealed in this work is considered as one of the mechanisms for self-incompatibility [40]. The self- incompatibility is one of the most important means for avoiding self-pollination and self-fertilization [42–44] as well means for preventing inbreeding, and for promoting the generation of new genotypes in plants. It is one of the dominant reasons for the spread and success of the angiosperms on earth [42–44]. The revealed features of the male and female generative sphere, as tenuinucellate ovule with one-celled archesporium, differentiation of specialized structures enabling the trophic of the embryo sac (integumentary tapetum, postament) are signs of high specialization of the generative sphere [40]. The lack of apomixis shows an adaption to the environmental conditions that are specific for each plant species. The ability of species to reproduce asexually along with the sexually one (facultative apomicts) increases its adaptability and expands its range of distribution. Hojsgaard at al. [45] pointed out that the facultative apomixis in perennials provides long-term reproductive stability for the colonization of large areas. In contrast, the lack of apomixis (when the species is strictly sexually reproductive) diminishes its adaptability and makes it dependent on environmental conditions. Therefore, we consider that the lack of apomixis is one of the probable causes for adherence to particular habitats and the limited distribution of the studied species. We can assume that the observed genetic variation and the established stability of the processes in the male and female generative spheres indicate good gene flow between individuals. Despite the theoretical prediction, that fragmenting small populations of species may exhibit low levels of genetic diversity, due to fragmentations and its a negative effect on pollination and on plant reproduction [46]. The established moderate level of genetic variation, unusual for an endemic, indicates a lack of genetic bottleneck in this species. This result was also supported by AMOVA where a high level of intrapopulation variation was observed (Figs 1 and 2). The genetic values found in our study might represent historic genetic diversity, derive from an ancestral population. In order of this, the size of *Alkanna* populations has been dramatically reduced in the last years, and the present habitats are highly fragmented. In all existed localities, population size is limited to several plants and the exchanges of pollen between individuals is highly restricted. This process will reduce the long term populations adaptability to present changing environmental conditions. This basic knowledge will help to determine the vulnerability and the resilience of important Bulgarian terrestrial ecosystems, with respect to global change factors such as potential increased temperatures and drought periods. More specifically, the results from this study may contribute to the development of management strategies for the protection and conservation of natural habitats such as the ecosystem community dominated by *Alkanna* species.

Pyrrolizidine alkaloids (PAs) are important secondary metabolites synthesized and accumulated in Boraginaceae, including plants in genus *Alkanna* [13,47]. The PAs are known to be toxic compounds that can alkylate DNA and thus cause mutations and even cancer in humans and in grazing animals [14]. Despite the toxicity of these alkaloids, plants synthesizing and accumulating them are of industrial interest for the pharmaceutical industry for potential new drug development [48]. *Alkanna tinctoria* has been the most extensively studied of the species in genus *Alkanna* and it was previously reported to contain PAs (7-*A*ngeloylretronecine, 9-angeloylretronecine, 7-tigloylretronecine, 9-tigloylretronecine, 7-seneioylretronecine, 9-senecioylretronecine, triangularine, dihydroxytriangularine, triangularicine, dihydroxytriangularicine, 7-angeloyl-9-(hydroxypropenoyl) retronecine, 7-tigloyl-9-(hydroxypropenoyl) retronecine, 7-angeloyl-9-(2,3-dihydroxypropanoyl) retronecine, 7-tigloyl-9-(2,3-dihydroxypropanoyl) retronecine) [13,34]; some of these alkaloids were found in the three endemic *Alkanna* species subject to this study. This study found eight alkaloids in *A*. *primuliflora* and *A*. *graeca* and six alkaloids in *A*. *stribrnyi* (Table 5). The main alkaloid in all investigated species was triangularine (5). *Alkanna primuliflora* and *A*. *graeca* showed similar alkaloid composition, whereas in *A*. *stribrnyi* the alkaloids triangularicine (**6**) and dihydroxytriangularicine (**8)** were not found (Table 5).

Because the metabolite patterns of the genus are influenced by the environmental conditions, the alkaloid profile of *A*. *stribrnyi* was investigated from three various natural localities (Table 1). The observed differences on the alkaloid content of *A*. *stribrnyi* (Ast1, Ast2 and Ast3) are minimal, mainly concerning the alkaloids 9-tigloylretronecine (**4**) and dihydroxytriangularine (**7**) (Table 5). There were no differences in alkaloid profile in the two stages of development of *A*. *primuliflora* (Aprm1, Aprm2) (Table 5). The environment has a little influence on the alkaloid biosynthesis in the genus.

## 5. Conclusions

Conservation of biodiversity is closely linked to the genetic diversity and reproductive capacity of species. The population genetic structure of *A*. *primuliflora*, *A*. *stribrnyi* and *A*. *graeca* showed a clear differentiation between the three species. Most probably, the genetic diversification of the populations are very high and has likely been limited by the ecological factors and habitat specialization. However, these factors have little influence on the alkaloid biosynthesis in the genus, and the three *Alkanna* species showed similar alkaloid patterns. The pyrrolizidine alkaloids were established for the first time in *A*. *primuliflora*, *A*. *graeca* and *A*. *stribrnyi*. The peculiarities of the reproductive biology of the three *Alkanna* species revealed them as sexually reproducing species and showed similarities between them. The successful development of the reproductive sphere (male and female) of studied species is the guarantee for the formation of seeds. The seed formation is very important for the dispersal of the species and the population size, and to carry out both *in-situ* and *ex-situ* conservation for these species. This study also observed the limited number of the three *Alkanna* species in their natural populations. The *in-situ* and *ex-situ* activity are important actions for the conservation of the endangered plant species. The authors will make the results available to the Ministry of Environment and Water of Bulgaria. These endemic species and their populations need to be considered for protection status.

## Abbreviations

Aprm1: *A*. *primuliflora* in flowering
Aprm2: *A*. *primuliflora* after flowering
Ast1: *A*. *stribrnyi*
GC/MS: Gas chromatography–mass spectrometry
ISSR: Inter-simple sequence repeats
PAs: pyrrolizidine alkaloids
TLC: thin layer chromatography
